# Multiscale computing for science and engineering in the era of exascale performance

**DOI:** 10.1098/rsta.2018.0144

**Published:** 2019-02-18

**Authors:** Alfons G. Hoekstra, Bastien Chopard, David Coster, Simon Portegies Zwart, Peter V. Coveney

**Affiliations:** 1 Computational Science Laboratory, Institute for Informatics, Faculty of Science, University of Amsterdam, The Netherlands; 2 High Performance Computing Department, ITMO University, St Petersburg, Russia; 3 Department of Computer Science, University of Geneva, Switzerland; 4 Institute for Plasma Physics, Garching, Germany; 5 Leiden Observatory, Leiden University, The Netherlands; 6 The Centre for Computational Science, Department of Chemistry, University College London, UK

**Keywords:** multiscale modelling and simulation, multiscale computing, exascale

## Abstract

In this position paper, we discuss two relevant topics: (i) generic multiscale computing on emerging exascale high-performing computing environments, and (ii) the scaling of such applications towards the exascale. We will introduce the different phases when developing a multiscale model and simulating it on available computing infrastructure, and argue that we could rely on it both on the conceptual modelling level and also when actually executing the multiscale simulation, and maybe should further develop generic frameworks and software tools to facilitate multiscale computing. Next, we focus on simulating multiscale models on high-end computing resources in the face of emerging exascale performance levels. We will argue that although applications could scale to exascale performance relying on weak scaling and maybe even on strong scaling, there are also clear arguments that such scaling may no longer apply for many applications on these emerging exascale machines and that we need to resort to what we would call *multi-scaling*.

This article is part of the theme issue ‘Multiscale modelling, simulation and computing: from the desktop to the exascale’.

## 1. Introduction

In science, our goal is to convincingly explain the processes at work in phenomena that we observe, as well as to predict what will occur before it does so. Predictions of real-world events all need substantial quantities of data and validated computational models together with the execution of many high-fidelity simulations. In many cases, the models that describe the phenomena are multiscale, as their accuracy and reliability depend on the correct representation of processes taking place on several length and time scales. Multiscale phenomena are everywhere around us [1–7]. If we study the origin and evolution of the Universe [8,9] or properties of materials [10–14], if we try to understand health and disease [3,15–22] or develop fusion as a potential energy source of the future [23], in all these cases and many more we find that processes on quite disparate length and time scales interact in strong and nonlinear ways. In short, multiscale modelling is ubiquitous and progress in most of these cases is determined by our ability to design and implement multiscale models of the particular systems under study [1,6,24,25].

The increasing importance of multiscale modelling in many domains of science and engineering is clearly demonstrated in numerous publications (e.g. [1,26]). Therefore, we must anticipate that multiscale simulations will become an ever-more important form of scientific application on high-end computing resources, necessitating the development of sustainable and reusable solutions for such applications. That is, we expect to need generic algorithms for multiscale computing.

We therefore require innovative new ways of computing to face the challenges posed both by multiscale modelling and simulation and by the emerging high-end computing ecosystem [27]. This will contribute to our ability to solve multiscale problems and, as we will argue, can *also* offer an avenue for new ways to efficiently exploit exascale resources. Multiscale computing could face these challenging by deploying its various single-scale components across heterogeneous architectures of exascale resources, mapped to produce optimal performance and designed to bridge both temporal and spatial scales [28–31]. Therefore, we should embark upon a programme to efficiently deploy multiscale codes on today's and future high-performance computers and, thereby, establish a new and more effective paradigm for exploiting current and emerging computing resources.

In this position paper, we explore and discuss generic multiscale computing on emerging exascale high-performing computing (HPC) environments. We will first discuss the different phases when developing a multiscale model and simulating it on available computing infrastructure, and analyse where in our view we could, and maybe should, continue to further develop generic frameworks and software tools to facilitate multiscale computing. Next, we will focus on simulating multiscale models on high-end computing resources, which we call High Performance Multiscale Computing (HPMC), in the face of emerging exascale performance levels. We will argue that strong *and* weak parallel scaling of monolithic applications often may reach its limits at the exascale and that we need to invoke what we would call *multi-scaling*. Note that although our analysis is driven by the needs of modelling multiscale systems, our arguments with respect to the scalability challenge for multiscale systems to the exascale also point to the necessity to consider new approaches to increase concurrency within complex (single-scale) models through new algorithms and corresponding implementations.

## 2. Generic multiscale modelling and simulation

### (a) Simulation-based science

Simulation-based science is all about formulating computational models of phenomena that we observe, and performing computer simulations in order to deepen our understanding of the systems that underpin these phenomena. The aim is to predict their future behaviour or to find adaptations that would change their behaviour in some desired way. Simulation-based science is sometimes called the third pillar of science and complements theory and experiments. Together they underpin the scientific method and strongly interact. Theory provides the necessary framework for computational models, experiments provide the data against which the computational models need to be validated, and numerical simulations may lead to new insights and theory or new hypotheses that are tested experimentally.

**Figure 1.**
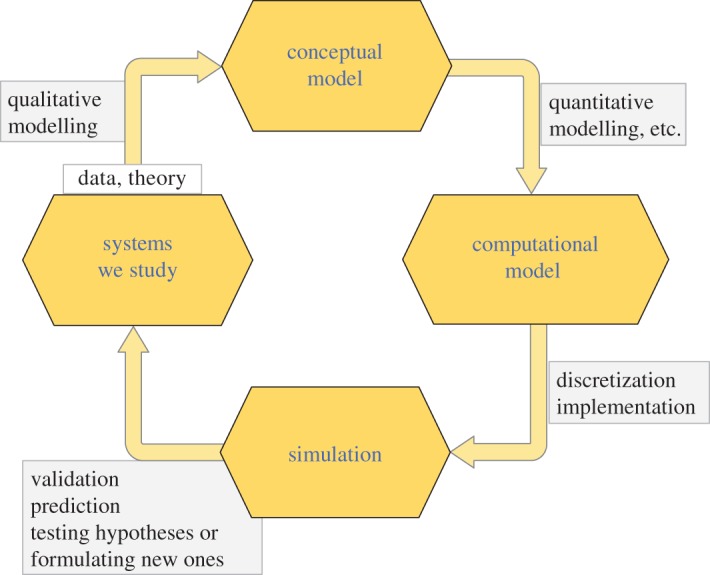
The modelling and simulation cycle. (Online version in colour.)

In performing simulation-based science, we usually go through a generic modelling and simulation cycle (figure 1). Based on available (observational) data and knowledge and theory of the phenomenon under study, a conceptual model is formulated, which is then turned into a computational model. This is then implemented on a computer after which we perform numerical experiments with the computational model. These simulations provide results that are used to validate our models and, once validated, to predict the behaviour of the system we study.

The power of this approach lies in the fact that the computational sciences have developed a large collection of well-established generic modelling paradigms (such as ODE or PDE-based methods, particle-based methods, agent-based methods, fully discrete methods, etc.) and a large collection of well-established (numerical) methods to discretize these computational models and implement them very efficiently on the full range of available computers (from the laptop via cloud to petaflop/s supercomputers). We argue that also in multiscale modelling and simulation such generic approaches are possible and have been demonstrated in a number of successful projects. Having been exposed to such solutions over the last decade, we will discuss the potential of generic multiscale modelling and simulation, with emphasis on high-end performance levels, projecting forward to the era of exascale computing.

### (b) The multiscale modelling and simulation framework

**Figure 2.**
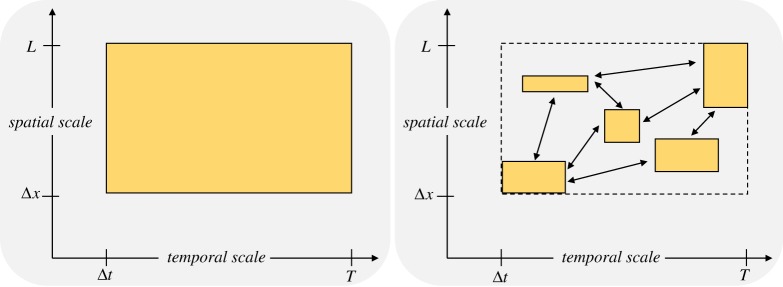
Decomposition of a monolythic application covering many spatial and temporal scales into several coupled single-scale submodels. (Online version in colour.)

Many applications in computational science involve large ranges of spatial and temporal scales. Multiscale modelling amounts to splitting the spatio-temporal scales into what are often called single-scale submodels. These submodels are then coupled through various scale-bridging techniques. Such a multiscale model, that is, a collection of single-scale submodels coupled via scale bridging algorithms, should then be a sufficiently accurate representation of the behaviour of the problem where all the scales are present, yet with a substantial reduction of computing needs. Figure 2 illustrates the multiscale approach as defined in the Multiscale Modelling and Simulation Framework (MMSF) [28,32].

**Figure 3.**
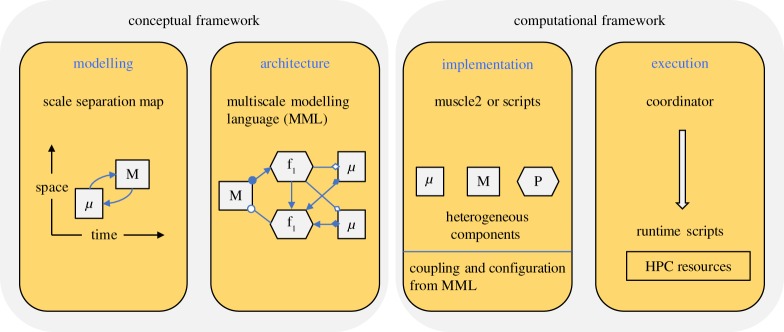
The multiscale modelling and simulation framework. (Online version in colour.)

The MMSF is a theoretical and practical method for modelling, characterizing and simulating multiscale phenomena. The MMSF has been developed and applied over the past 10 years [1,28,29,31,33–35], and comprises a four-stage pipeline from developing a multiscale model to executing the multiscale simulation. This is shown in figure 3. First, we model a phenomenon by identifying relevant processes that are well described by single-scale submodels, and their relevant scales, using the Scale Separation Map (SSM, the right panel in figure 2 is an example of an SSM). The architecture of the multiscale model, that is the communication between the single-scale models and details of the scale-bridging methods, are then specified in the Multiscale Modelling Language (MML) [36]. This specification is then used to (semi) automatically glue the single scale components (software implementations of the single-scale models) and the scale bridging components together using some dedicated coupling toolkit (such as, for instance, Muscle [33,37], MaMiCo [38] or AMUSE [9,39]). Finally, the multiscale simulation can be executed, by using dedicated environments such as, e.g. QCG [40]. This can in principle be done in a highly automated, optimized way, certainly when targeting HPC infrastructure [31,41].

### (c) Generic frameworks

From the start of the development of the MMSF and comparable frameworks for multiscale modelling and simulation (for an overview, see the paper by Groen *et al.* [42] in this special issue), the ambition has always been to be as generic as possible, in the sense that such environments should be applicable for a large range of applications from different domains, that software components (implementing single-scale models or e.g. scale bridging) be reusable and interchangeable, and relieve the users as much as possible of the intricacies of launching and executing complex multiscale models on equally complex distributed HPC infrastructure.

We argue, based on a range of projects in multiscale modelling and computing, that the four phases of the MMSF (figure 3) are generic and follow the simulation-based science cycle from figure 1. The Scale Separation Map and its more detailed description encoded in the MML together form the Conceptual Multiscale Model, whereas the full implementation of the single-scale models, the scale-briding algorithms, glued together according to the MML architecture using coupling frameworks, forms the Computational Multiscale Model. The final execution phase in the MMSF is then the multiscale simulation.

This observation would warrant generic all-encompassing software environments to define a conceptual model, which is then translated by this environment into a computational model, and is finally executed on a range of computing infrastructures, again facilitated by such generic environments. Although this is certainly possible, as demonstrated, e.g. by our own developments [32], it is however not surprising that a range of different environments have been developed to, e.g. connect single codes into a full multiscale simulation. Usually, this is within specific communities, e.g. weather/climate [43], fusion [23] or astrophysics [44]. These environments run in production and are tailored to the needs of their communities.

This then triggers the question as to what the benefits of generic frameworks could be, if any, and where they could be most useful. We believe that such frameworks should lead to a separation of concerns, where the user can focus on the modelling and simulation itself. A main ingredient in multiscale modelling is coupling single-scale models using scale bridging to convert information from one scale to another. Such coupling increases the complexity of multiscale modelling and simulation, and software should help mitigate that, both on the conceptual level and the simulation level. Moreover, generic frameworks should allow the reuse of validated and tested modules (submodels) and this could help in effectively standardizing data formats.

**Figure 4.**
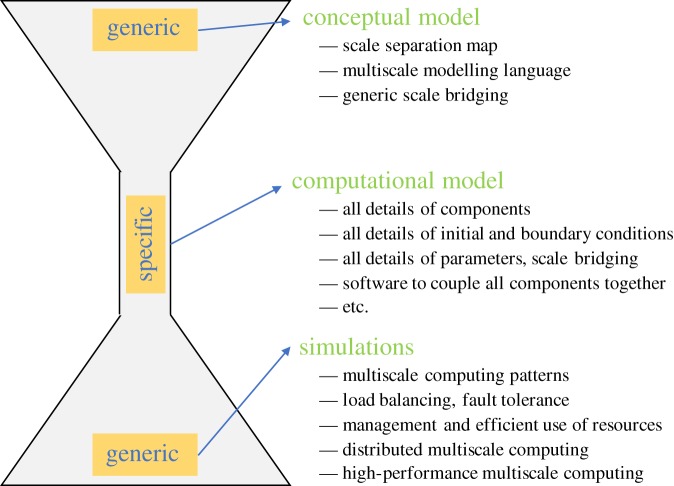
The multiscale computing hourglass. (Online version in colour.)

In our opinion, generic tools could be beneficial to the users both on the level of developing a conceptual multiscale model and on the level of executing the multiscale model. We call this the multiscale computing hourglass (see figure 4). It is important to continue to develop frameworks for multiscale computing on both levels, as we will argue below in more detail.

In MMSF, a conceptual multiscale model is defined as a collection of single-scale models, their connection including scale-bridging methods, and the details of the connection scheme [28]. The MML provides a formal way to specify the conceptual multiscale model [28,36], and using its xml version called xMML, it can be fully specified in a machine-readable text file. Such formal specification, in the form of an xMML file, could then be input to the neck of the hourglass in figure 4, where actual ‘glue’ code needs to be implemented. The benefits of agreeing on a formal specification of a multiscale model, relying, e.g. on MML, should not be underestimated and will bring many benefits. First, it allows clear and well-defined communication with colleagues, as well as the sharing of multiscale models. One could, for instance, imagine repositories of xMML files, very much like existing repositories of SBML [45] or CellML [46–48] models. It could also form the starting point for a mathematical framework to reason about properties of a multiscale model, ranging from proving that it does not contain a deadlock [28], via using it as input for multiscale uncertainty quantification methods [49], to estimating and optimizing its computational footprint [31].

The neck of the hourglass contains the actual creation of the multiscale computational model, where all the application-specific details need to be established (figure 4). One important component is software to glue together the single-scale components using some coupling toolkit. Although some of these toolkits have been developed to be again generic and suitable across scientific domains (e.g. Muscle [33,37]), most coupling libraries used in production today are developed and maintained in specific scientific communities, e.g. astrophysics [39], complex fluids [38,50], quantum chemistry [51] or climate modelling [43]. Although we would very much welcome an exchange of ideas and technologies between these communities, to avoid re-inventing the wheel, we also acknowledge that given the current state of the art, it would be unrealistic to push for generic coupling toolkits and environments, comparable to, e.g. MPI in parallel computing.

What would be profitable is to link generic concepts for conceptual multiscale modelling, as laid down in xMML files, to these coupling environments. For instance, one could compile xMML files into ‘glue code’ and ‘wrapper code’ for a specific environment, as we have demonstrated with Muscle [28,33]. Such technology would mitigate much of the aforementioned complexity in relation to coupling together single-scale components and scale-bridging code snippets. In our experience, such high level inter-operability does not necessarily induce overheads (e.g. [29]), and even if so, we believe that the benefits in terms of maintainability and extensibility of complex multiscale models would outweigh such trade-offs. Moreover, this approach also allows for straightforward ‘plug-and-play’, where, for instance, different implementations of a single-scale model, that may perform optimally on different architectures, are quickly interchanged.

Finally, at the bottom of the hourglass, when simulating the multiscale model as efficiently as possible on available computing resources, we find again ample room for generic solutions that would alleviate the burden of mapping a complex multiscale simulation in the most efficient way to hardware. Certainly when the targeted hardware consists of HPC machines, distributed systems, cloud environments or a combination of all these, and when issues like load balancing, advanced reservations, fault tolerance and energy efficiency need to be taken into account and optimized, we believe that generic solutions, to which the coupling libraries discussed above could interface, would be highly beneficial.

To do so we have proposed the concept of Multiscale Computing Patterns (MCPs), which are generic and recurring call sequences at the level of the single-scale components of a multiscale simulation [31]. It is beyond the scope of this manuscript to discuss the details of MCPs. It suffices to note that we proposed three such patterns, which capture most, if not all, multiscale simulations. We have proposed and implemented a pilot of MCPs software that takes as input the generic xMML description of a multiscale model, in combination with performance measurements of the components that make up the multiscale model (both in terms of computational performance and energy usage on massively parallel machines), and then interfaces with middleware, such as in our case QCG [40], to propose an optimal mapping of the multiscale simulation to available hardware, and to schedule and finally execute the simulation [41]. Note that such a generic deployment of the multiscale simulation only requires knowledge of the control structure of the multiscale model, as available via the xMML, and detailed knowledge of the performance of the single-scale models (both in terms of execution time, but also in terms of e.g. energy usage). If this is available for a range of (heterogeneous) architectures, the MCPs software can select, in a generic way and for each type of pattern, an optimal way to perform the multiscale simulation, given available hardware and even considering expected queuing times for that hardware [41]. Although these are very recent developments, in our view such generic solutions for scheduling and executing multiscale simulations on complex computing ecosystems consisting of large HPC machines, distributed environments and clouds, and interfacing these solutions to the most popular multiscale coupling libraries and to the most relevant scheduling environments and queuing systems, could be extremely beneficial to users, who no longer need to worry about this time-consuming and complex phase of multiscale computing.

## 3. Towards the exascale

### (a) Strong and weak scaling

As the clock speeds of individual cores are no longer increasing, emerging exascale machines can only reach their anticipated exaflop/s computational speeds by aggregating larger and larger numbers of nodes and cores. As a result, these machines are becoming ‘fatter’, not faster. We have argued before that as a result of this we are approaching the limits of what is achievable using monolithic codes [31]. Our argument was that ‘because the parallelism is usually applied to the spatial domain, we are increasingly simulating larger slabs of matter and bigger chunks of the Universe, applying weak scaling by using more particles, a higher grid resolution or more finite elements. Yet it often is the temporal behaviour that one is really interested in, and that behaviour is not extended by adopting larger computers of this nature, or by making the problem physically larger. Since the scientific problems of interest usually have time scales which scale as a nonlinear function of the volume of the system under investigation, each temporal update requires more wall clock time for larger physical problems. This is in fact a recipe for disaster: we are not getting closer to studying large space and long time behaviour with monolithic codes’. We consider this to be a cogent argument to suggest that monolithic codes could be the exception on exascale machines, as weak scaling may not produce the desired results. If so, we should invest in developing other scenarios, including high-performance multiscale computing, or scenarios where monolithic codes (or coupled multiscale codes for that matter) are repeated over and over again in ensembles, e.g. in parameter sweeping scenarios or for uncertainty quantification (see also Portegies Zwart, who recently made a similar argument [27]).

We will now further analyse this argument by looking in detail at different scaling scenarios, relying on the Scale Separation Map as introduction in figure 2 and high-level modelling of parallel performance.

**Figure 5.**
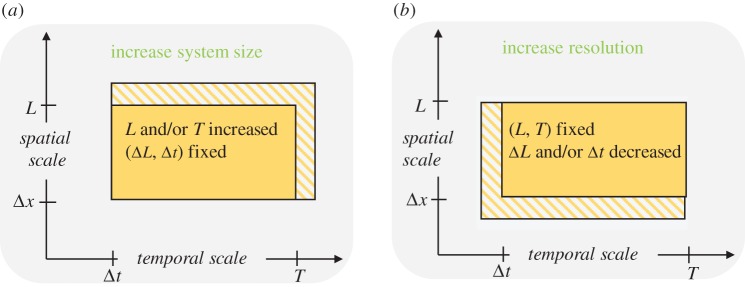
Weak scaling scenarios. (*a*) By increasing system size and simulated time and (*b*) by increasing temporal and spatial resolution. A combination of both is also possible. (Online version in colour.)

If we would assume that we can continue using emerging exascale machines efficiently by relying on weak scaling, we basically hope that much larger systems can be simulated. And that this is possible without new additional algorithmic strategies, but by just relying on the huge additional computing power offered by such exascale machines. Figure 5 shows different scaling scenarios on a scale map. For instance, in climate modelling, the system size *L* is likely to stay the same (the full Earth), as well as the time span of the simulation *T*. But accuracy may be improved, which requires reductions in Δ*t* and Δ*x*. Decreasing Δ*t* would imply more iterations to reach the time span *T*, and therefore our argument holds.

For other applications, *T* might already be sufficient, but *L* needs to be increased. As an example, one may consider the transport of sediments in a river over a given time period. The capability to consider a longer stretch of the river is important. Also, when computing blood flow, we may want to add more vessels in the simulation, thus increasing *L*. In this case, the argument above would not hold and weak scaling remains a good option.

It is likely, however, that both *L* and *T* need to be increased in proportion because phenomena at a larger time scale usually also imply a larger spatial scale. It is in such scaling scenarios, which we believe are most common, where we may be reaching the limits of what is achievable by just increasing the number of processors, as we need to do in order to reach exascale performance levels.

We will now explore the limits of increasing *L* and/or *T*, assuming that a much larger computer becomes available. We will show that for some applications exascale machines can bring a substantial gain, but for others scaling is limited, and new multiscale techniques need to be developed to exploit the additional computing power. Note that here we mainly focus the discussion on increasing *L* or *T*, but the same approach can be used to study the possibility to decrease Δ*t* or Δ*x* to improve accuracy.

Let us consider a parallel code whose execution time *T*_par_ reads

3.1 $$T_{\mathrm{par}}=\frac{W}{pR}+\Omega (W,p)=\frac{W}{pR}\left[1+\frac{pR\Omega }{W}\right],$$

where *W* is the total work (e.g. the number of operations required to solve the problem), *p* the number of cores, *R* the speed of the core (number of operations per second) and *Ω* the overhead resulting from the parallelization. The efficiency *E* is then

3.2 $$E(W,p)=\frac{T_{\mathrm{seq}}}{pT_{\mathrm{par}}}=\frac{W/R}{W/R+p\Omega }=\frac{1}{1+pR\Omega /W}.$$

Note that the quantity *pRΩ*/*W* is the total fractional overhead [52]. In all generality, we can write

3.3 $$T_{\mathrm{par}}=\frac{W}{pRE(W,p)}$$

showing that the effective power of the processors is reduced by a factor equal to the efficiency of the parallel implementation.

The relation *W* = *W*(*p*) that guarantees that *E* stays constant is called the isoefficiency function. Weak scaling is achieved by increasing *W* as *p* increases, typically according to the isoefficiency relation. Strong scaling is achieved by increasing *p* while keeping *W* constant. In the former case, one solves a larger problem on more processors, within about the same time *T*_par_. In the latter case, one expects to solve a given problem faster by using more processors.

For instance, for an iterative stencil-based calculation on a three-dimensional mesh, one has a communication overhead between each subdomain, at each iteration. Let us assume that we have *n* iterations and that *w* = *W*/*n* is the work per iteration, which is proportional to the sum of the volumes of the subdomains. The communication overhead is then proportional to the boundary of one subdomain, namely to (*W*/(*np*))^2/3^ = (*w*/*p*)^2/3^. Thus,

3.4 $$T_{\mathrm{par}}=\frac{W}{pR}+nC{\left(\frac{w}{p}\right)}^{2/3},$$

where *C* is a constant depending on the speed of the interconnection network. Thus *Ω* = *nC*(*w*/*p*)^2/3^ and

3.5 $$E=\frac{1}{1+pR\Omega /W}=\frac{1}{1+RCp{\left(w/p\right)}^{2/3}/w}=\frac{1}{1+RC{\left(p/w\right)}^{1/3}}.$$

The isoefficiency function is then *W*(*p*)∼*p*.

Many scientific applications correspond to the time evolution of a spatial quantity. The typical spatial and temporal scales that can be resolved depend on the space and time discretization Δ*x* and Δ*t*, as well as *L*, the spatial extension of the system, and *T*, the duration of the simulation. Spatial and temporal scales outside this interval will not be resolved. One can estimate the CPU time required for such a simulation. The total work *W* is typically

3.6 $$W=\beta {\left(\frac{L}{\Delta x}\right)}^{d}\left(\frac{T}{\Delta t}\right),$$

where *β* is a proportionality factor and *d* is the spatial dimension. The number of iterations is *n* = *T*/Δ*t* and the work per iteration is

3.7 $$w=\beta {\left(\frac{L}{\Delta x}\right)}^{d}.$$

If we again assume a stencil-based calculation on a three-dimensional mesh, combining equations (3.3), (3.5)–(3.7) results in

$$T_{\mathrm{par}}=\left[\frac{\beta }{R}{\left(\frac{L}{\Delta x}\right)}^{3}p^{-1}+\beta^{2/3}C{\left(\frac{L}{\Delta x}\right)}^{2/3}p^{-2/3}\right]\left(\frac{T}{\Delta t}\right).$$

We can write this as

3.8 $$T=\frac{pT_{\mathrm{par}}\Delta t}{\left[(\beta /R){\left(L/\Delta x\right)}^{3}+\beta^{2/3}C{\left(L/\Delta x\right)}^{2/3}p^{1/3}\right]}.$$

The above equation relates the physical time *T* that can be simulated within a parallel execution time *T*_par_ as a function of the spatial extension *L* of the system and the number of processors *p*. This equation *T* = *T*(*L*) can be seen as an iso-performance relation, or an iso-*T*_par_ curve.

Let us now take specific values for the performance model given in equation (3.8) (table 1). Here we assume a Lattice Boltzmann (LB) simulation^1^ of blood flow in an aneurysm of centimetre size, resolved at 10 μm, for two heart beats. Time resolution is 10^−5^ s, and we assume cores able to perform 0.4 × 10^9^ double precision operations per second. A typical LB iteration requires *β* = 200 arithmetic operations and the exchange of typically 20 populations of 8 bytes. Thus, we set *C* = 1 GB s^−1^ × 20 × 8 = 1.5 × 10^−7^.

**Table 1.** Specific values for LB example.

*R* (s^−1^)	*C* (s)	*β*	*p*	*T*_par_ (day)	*L* (m)	Δ*x* (m)	Δ*t* (s)	*T* (s)
0.4 10^9^	1.5 10^−7^	200	1000	1.5	0.01	10^−5^	10^−5^	2

**Figure 6.**
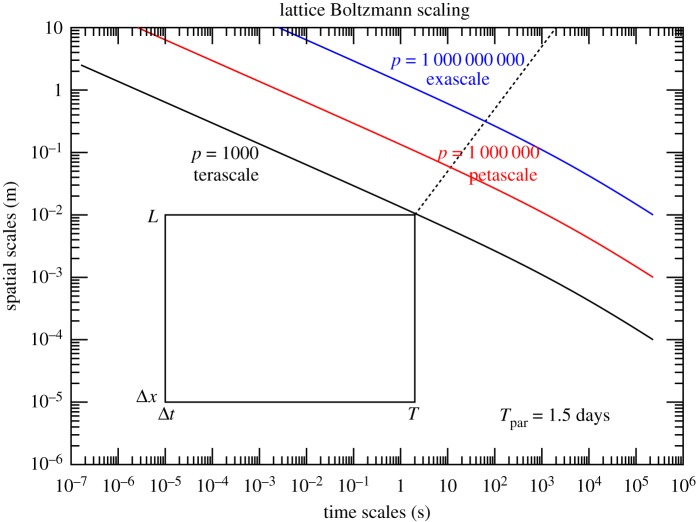
Scaling of a lattice Boltzmann code at different performance levels. See text for a detailed account of the behaviour shown here.

Figure 6 show the iso-performance curve (3.8), assuming that we consider a computation of *T*_par_ = 1.5 days. These curves indicate how *L* and *T* can be varied for the same *T*_par_, while keeping Δ*x* and Δ*t* fixed.

When getting a faster machine, with *m* times more processors, we see that we can consider the same physical time *T*, and increase the system size by the expected factor *m*^1/3^. This is weak scaling. On the other hand, one can also keep the same system size and increase the simulation time *T*. This corresponds to a strong scaling situation, except that here we keep *T*_par_ = 1.5 days constant and compute a larger time scale. As we can see from figure 6, the potential is less significant, although still interesting. For instance, taking *p* = 1 000 000 and the same *L* allows us to reach *T* = 10^3^ s, that is an increase of a factor 500, with respect to 1000 times more processors. With *p* = 1 000 000 000, the value of *T* becomes 2 × 10^5^, hence an increase of a factor 10^5^ with 10^6^ additional processors. We also notice that these iso-curves stop when *L* becomes too small, corresponding to the situation where each processor would hold less than one data element.

It is, however, usually more likely that both *L* and *T* need to be increased. Typically, when analysing convective processes, *L* and *T* must grow proportionally (for a diffusive process, *L* would grow as $\sqrt{T}$). Figure 6 shows with a dotted line an increase of *L* and *T* by the same factor (up to a 1000). Its intersection with the exascale iso-performance line gives *T* = 8 × 10^1^ s and *L* = 4 × 10^−1^ m. This is a 40 times longer simulation time, for a system 40 times larger, thus with 40^3^ = 64 000 more mesh points. Therefore, if such a constraint holds between *L* and *T*, the possibility to reach larger physical time scales is very limited, even with a one million-fold performance increase. This is in line with our original qualitative argument that weak scaling starts to break down if we only increase processor numbers without increasing processor speeds.

Next we consider another application that scales less well than LB models. We assume an N-body problem with long-range forces. We consider a $O(N^{2})$ implementation, assuming that the Barnes–Hut algorithm does not apply here because the particles are on average too close to each other. The sequential execution time is

3.9 $$T_{\mathrm{seq}}=\left[\frac{11}{R}N(N-1)+\frac{6}{R}N\right]\frac{T}{\Delta t},$$

where the first term corresponds to the computation of the pairs interaction forces (e.g. gravity needs 11 operations) and the second is the time integration (e.g. Verlet requires six operations). *R* is the speed of a core, *T*/Δ*t* is the number of time steps.^2^

With *p* cores and *N*/*p* particles per core, the force computation requires an *all-to-all* communication, which can be realized with *p* − 1 communications involving all cores *P*_*i*_ in parallel. At stage *k*, *k* = 1, …, *p* − 1, *P*_*i*_ sends 3*N*/*p* coordinates to *P*_*i*+*k*_ modulo *p*. The resulting communication time is

3.10 $$T_{\mathrm{comm}}=3\times 8\times C\times (p-1)\times \frac{N}{p},$$

because there are three double precision spatial coordinates per particle. *C* is the time to exchange a byte between two cores.

Thus, the parallel time *T*_par_ is

3.11 $$T_{\mathrm{par}}=\left[\frac{11N}{pR}(N-1)+\frac{6N}{pR}+24C(p-1)\frac{N}{p}\right]\frac{T}{\Delta t},$$

and, from this relation, we obtain

3.12 $$\frac{T}{\Delta t}=\frac{pT_{\mathrm{par}}R}{11N(N-1)+6N+24CR(p-1)N}.$$

**Figure 7.**
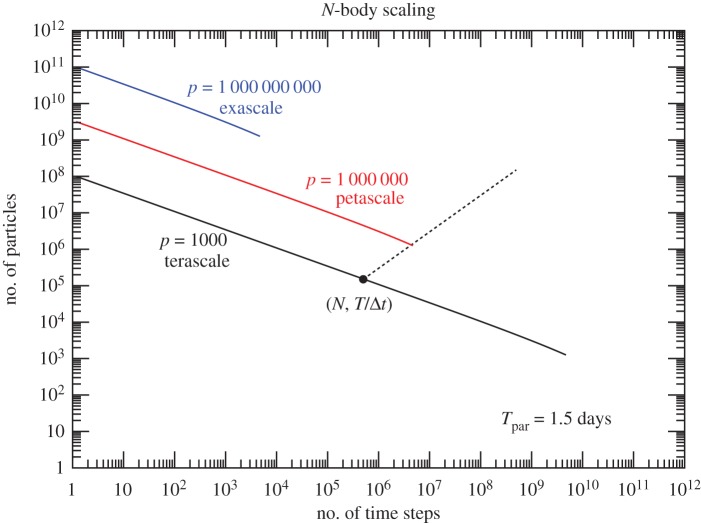
The relationship between the number (*N*) of particles and the number (*T*/Δ*t*) of iterations that are possible within *T*_par_ = 1.5 days, and different numbers of processors (*p*).

This relation links the number of iterations *T*/Δ*t* that are possible with *N* particles within a computational time *T*_par_. We call this relation the *T*_par_ iso-line. Following the same approach as in the previous case, we show in figure 7 this iso-line for different scales of computing resources. Here, we took *R* = 10^9^ s^−1^, *C* = 10^9^ s. The *T*_par_ iso-lines stops when the number of particles per core reaches 1. From figure 7, we see first that adding more cores allows one to consider more particles, but not in proportion to the increase of computing power. Second, the possibility of running the simulation to larger physical time is rather limited. And worse, the maximum possible number of iterations may even decrease as the number of cores increases, as is the case for the reference simulation (*N*, *T*/Δ*t*). This behaviour is obviously due to the poor scaling of an *all-to-all* communication and suggests that the exascale may not allow one to solve larger N-body problems with a simple brute force approach.

These results indicate that some single-scale monolithic applications (e.g. lattice Boltzmann solvers) still have good potential to exploit exascale computers, but for others (e.g. N-body simulations) exascale may not bring any benefit to simulate larger systems for a longer physical time. Smarter than brute force approaches are thus needed, such as for instance multiscale simulation where scales are separated within different sub-models.

### (b) Multi-scaling

**Figure 8.**
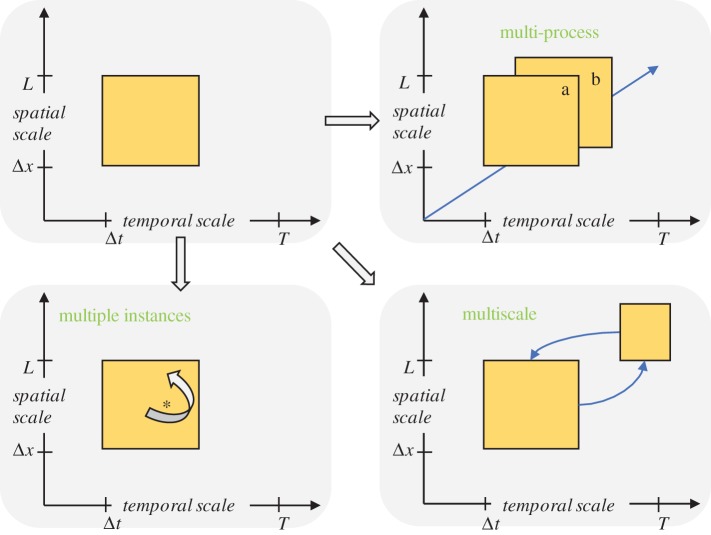
‘Multi-scaling’ for parallel performance, by adding more processes (upper right, multi-process), by executing a single-scale process multiple times in parallel (lower left, multiple instances) or by building a multiscale model (lower right), or any combination of these three. (Online version in colour.)

One way out of this dilemma is by taking highly efficient monolithic codes that perform well on the petascale, and combining them in loosely coupled ways to reach exascale performance. We call this *multi-scaling*, where ‘scale’ in this case does not refer to spatial or temporal scales, but to processor scales. We can identify a few scenarios for multi-scaling (figure 8).

One could run multiple instances of the code, e.g. in replica computing (parameter sweeping) applications or uncertainty quantification. One could also create a multi-process application, effectively coupling different processes together that overlap in spatio-temporal scales. Finally, one could create multiscale applications, coupling single-scale monolithic codes on different spatio-temporal scales. And, of course, combinations of these scenarios are possible, e.g. performing uncertainty quantification on a multiscale model [49].

It would be wise to explore further these types of applications and better understand how *multi-scaling* could help unleash the power of future exascale machines, but at the same time also face the challenges in relation to energy consumption and fault tolerance. We have already seen convincing examples, e.g. using the multiple instances approach, in replica computing for calculating binding affinaties [53,54].

## 4. Conclusion

Multiscale computing has turned into a mature paradigm, supported by many communities that have invested in production software. Yet, we see room for further development of generic solutions in conceptual multiscale modelling as well as in executing multiscale simulations. When seen in the light of the anticipated need for *multi-scaling* as opposed to weak scaling in order to reach exaflop/s performance, we conclude that such developments are very much needed for the optimal exploitation of future exascale HPC systems.

## Footnotes

1 The values of the parameters chosen here correspond to an actual run with Palabos, see www.palabos.org and www.thrombus-vph.eu.

2 Note that we do not assume advanced individual time-stepping algorithms in this example.

## Data accessibility

This article has no additional data.

## References

[1] Hoekstra AG, Chopard B, Coveney PV 2014 Multiscale modelling and simulation: a position paper. Phil. Trans. R. Soc. A 372, 20130377 (10.1098/rsta.2013.0377) 24982256

[2] Weinan E, Engquist B, Li X, Weiqing R, Vanden-Eijnden E 2007 Heterogeneous multiscale methods: a review. Commun. Comput. Phys. 2, 367–450.

[3] Sloot PMA, Hoekstra AG 2010 Multi-scale modelling in computational biomedicine. Brief Bioinform. 11, 142–152. (10.1093/bib/bbp038) 20028713

[4] Fish J 2009 Multiscale methods: bridging the scales in Science and Engineering. Oxford, UK: Oxford University Press.

[5] Engquist B, Lötstedt P, Runborg O 2009 Multiscale modeling and simulation in Science. Berlin, Germany: Springer.

[6] Karabasov S, Nerukh D, Hoekstra AG, Chopard B, Coveney PV 2014 Multiscale modelling: approaches and challenges. Phil. Trans. R. Soc. A 372, 20130390 (10.1098/rsta.2013.0390) 24982248 PMC4084530

[7] Coveney PV, Boon JP, Succi S 2016 Bridging the gaps at the physics chemistry biology interface. Phil. Trans. R. Soc. A 374, 20160335 (10.1098/rsta.2016.0335) 27698047 PMC5052737

[8] Portegies Zwart S *et al.* 2010 Simulating the universe on an intercontinental grid of supercomputers. IEEE Comput. 43, 63–70. (10.1109/mc.2009.419)

[9] Portegies Zwart S, McMillan S 2018 Astrophysical recipes: the art of AMUSE. Bristol, UK: IOP Publishers.

[10] Suter JL, Groen D, Coveney PV 2015 Chemically specific multiscale modeling of clay-polymer nanocomposites reveals intercalation dynamics, tactoid self-assembly and emergent materials properties. Adv. Mater. 27, 966–984. (10.1002/adma.201403361) 25488829 PMC4368376

[11] Silani M, Talebi H, Hamouda AM, Rabczuk T 2016 Nonlocal damage modelling in clay/epoxy nanocomposites using a multiscale approach. J. Comput. Sci. 15, 18–23. (10.1016/j.jocs.2015.11.007)

[12] Laurini E, Posocco P, Fermeglia M, Pricl S 2016 MoDeNa nanotools: an integrated multiscale simulation workflow to predict thermophysical properties of thermoplastic polyurethanes. J. Comput. Sci. 15, 24–33. (10.1016/j.jocs.2015.11.006)

[13] Bin S, Li Z 2016 Multi-scale modeling and trans-level simulation from material meso-damage to structural failure of reinforced concrete frame structures under seismic loading. J. Comput. Sci. 12, 38–50. (10.1016/j.jocs.2015.11.003)

[14] Suter JL, Coveney PV, Anderson RL, Greenwell HC, Cliffe S 2011 Rule based design of clay-swelling inhibitors. Energy Environ Sci. 4, 4572–4586. (10.1039/c1ee01280k)

[15] Anzai H, Ohta M, Falcone JL, Chopard B 2012 Optimization of flow diverters for cerebral aneurysms. J. Comput. Sci. 3, 1–7. (10.1016/j.jocs.2011.12.006)

[16] Coveney PV, Diaz-Zuccarini V, Graf N, Hunter P, Kohl P, Tegner J, Viceconti M 2013 Integrative approaches to computational biomedicine. Interface Focus 3, 737–738. (10.1098/rsfs.2013.0003)

[17] Garbey M, Rahman M, Berceli S 2015 A multiscale computational framework to understand vascular adaptation. J. Comput. Sci. 8, 32–47. (10.1016/j.jocs.2015.02.002) 25977733 PMC4426998

[18] Itani MA, Schiller UD, Schmieschek S, Hetherington J, Bernabeu MO, Chandrashekar H, Robertson F, Coveney PV, Groen D 2015 An automated multiscale ensemble simulation approach for vascular blood flow. J. Comput. Sci. 9, 150–155. (10.1016/j.jocs.2015.04.008)

[19] Kohl P, Viceconti M 2010 The virtual physiological human: computer simulation for integrative biomedicine II. Phil. Trans. R. Soc. A 368, 2591–2594. (10.1098/rsta.2010.0098) 20439263

[20] Omholt SW, Hunter PJ 2016 The Human Physiome: a necessary key for the creative destruction of medicine. Interface Focus 6, 237–243. (10.1098/rsfs.2016.0003)

[21] Paredes S, Rocha T, de Carvalho P, Henriques J, Mendes D, Cabete R, Bianchi A, Morais J 2015 The CardioRisk project: improvement of cardiovascular risk assessment. J. Comput. Sci. 9, 39–44. (10.1016/j.jocs.2015.04.025)

[22] Zasada SJ, Wang T, Haidar A, Liu E, Graf N, Clapworthy G, Manos S, Coveney PV 2012 IMENSE: An e-infrastructure environment for patient specific multiscale data integration, modelling and clinical treatment. J. Comput. Sci. 3, 314–327. (10.1016/j.jocs.2011.07.001)

[23] Falchetto GL *et al.* 2014 The European Integrated Tokamak Modelling (ITM) effort: achievements and first physics results. Nuclear Fusion 54, 043018 (10.1088/0029-5515/54/4/043018)

[24] Bruzzone AG 2015 Perspectives of modeling; applied simulation: modeling, algorithms and Simulations: advances and novel researches for problem-solving and decision-making in complex, multi-scale and multi-domain systems. J. Comput. Sci. 10, 63–65. (10.1016/j.jocs.2015.06.004)

[25] Stevens B, Bony S 2013 Climate change. What are climate models missing? Science 340, 1053–1054. 23723223 10.1126/science.1237554

[26] Groen D, Zasada SJ, Coveney PV 2014 Survey of multiscale and multiphysics applications and communities. Comput. Sci. Eng. 16, 34–43. (10.1109/MCSE.2013.47)

[27] Portegies Zwart S 2018 Computational astrophysics for the future. Science 361, 979–980. (10.1126/science.aau3206) 30190394

[28] Borgdorff J, Falcone JL, Lorenz E, Bona-Casas C, Chopard B, Hoekstra AG 2013 Foundations of distributed multiscale computing: formalization, specification, and analysis. J. Parallel Distrib. Comput. 73, 465–483. (10.1016/j.jpdc.2012.12.011)

[29] Borgdorff J *et al.* 2014 Performance of distributed multiscale simulations. Phil. Trans. R. Soc. A 372, 20130407 (10.1098/rsta.2013.0407) 24982258 PMC4084531

[30] Chopard B, Falcone JL, Kunzli P, Veen L, Hoekstra A 2018 Multiscale modeling: recent progress and open questions. Multiscale Multidisc. Model. Exp. Des. 1, 57–68. (10.1007/s41939-017-0006-4)

[31] Alowayyed S, Groen D, Coveney PV, Hoekstra AG 2017 Multiscale computing in the exascale era. J. Comput. Sci. 22, 15–25. (10.1016/j.jocs.2017.07.004)

[32] Chopard B, Borgdorff J, Hoekstra AG 2014 A framework for multi-scale modelling. Phil. Trans. R. Soc. A 372, 20130378 (10.1098/rsta.2013.0378) 24982249 PMC4084523

[33] Borgdorff J, Mamonski M, Bosak B, Kurowski K, Ben Belgacem M, Chopard B, Groen D, Coveney PV, Hoekstra AG 2014 Distributed multiscale computing with MUSCLE 2, the Multiscale Coupling Library and Environment. J. Comput. Sci. 5, 719–731. (10.1016/j.jocs.2014.04.004)

[34] Hoenen O, Fazendeiro L, Scott BD, Borgdorff J, Hoekstra AG, Strand P, Coster DP 2013 Designing and running turbulence transport simulations using a distributed multiscale computing approach. Mulhouse, France: European Physical Society.

[35] Belgacem MB, Chopard B, Borgdorff J, Mamonski M, Rycerz K, Harezlak D 2013 Distributed multiscale computations using the MAPPER framework. Procedia Comput. Sci. 18, 1106–1115. (10.1016/j.procs.2013.05.276)

[36] Falcone JLJL, Chopard B, Hoekstra A 2010 MML: towards a multiscale modeling language. Procedia Comput. Sci. 1, 819–826. (10.1016/j.procs.2010.04.089)

[37] Ben Belgacem M, Chopard B 2017 MUSCLE-HPC: a new high performance API to couple multiscale parallel applications. Future Gen. Comput. Syst. 67, 72–82. (10.1016/J.FUTURE.2016.08.009)

[38] Neumann P, Flohr H, Arora R, Jarmatz P, Tchipev N, Bungartz HJ 2016 MaMiCo: Software design for parallel molecular-continuum flow simulations. Comput. Phys. Commun. 200, 324–335. (10.1016/J.CPC.2015.10.029)

[39] Portegies Zwart S, Bedorf J 2016 Creating the virtual universe. IEEE Software 33, 25–29. (10.1109/MS.2016.113)

[40] Piontek T, Bosak B, Grabowski P, Kopta P, Kulczewski M, Szejnfeld D, Kurowski K 2016 Development of science gateways using QCG, lessons learned from the deployment on large scale distributed and HPC infrastructures. J. Grid Comput. 14, 559–573. (10.1007/s10723-016-9384-9)

[41] Alowayyed S *et al.* 2018 Patterns for high performance multiscale computing. Future Gen. Comput. Syst. 91, 335–346. (10.1016/j.future.2018.08.045)

[42] Groen D, Knap J, Neumann P, Suleimenova D, Veen L, Leiter K 2019 Mastering the scales: a survey on the benefits of multiscale computing software. Phil. Trans. R. Soc. A 377, 20180147 (10.1098/rsta.2018.0147) PMC6388006 30967042

[43] Valcke S *et al.* 2012 Coupling technologies for Earth System Modelling. Geosci. Model Dev. 5, 1589–1596. (10.5194/gmd-5-1589-2012)

[44] Bédorf J, Gaburov E, Fujii MS, Nitadori K, Ishiyama T, Portegies Zwart S, 2014 24.77 Pflops on a gravitational tree-code to simulate the Milky Way Galaxy with 18600 GPUs. In Proc. of the Int. Conf. for High Performance Computing, Storage and Analysis 2014, New Orleans, LA, 16–21 November, pp. 54–65. IEEE Press.

[45] Hucka M *et al.* 2003 The systems biology markup language (SBML): a medium for representation and exchange of biochemical network models. Bioinformatics. 19, 524–531. (10.1093/bioinformatics/btg015) 12611808

[46] Lloyd CM, Halstead MDB, Nielsen PF 2004 Cell ML: its future, present and past. Progress Biophys. Mol. Biol. 85, 433–450. (10.1016/J.PBIOMOLBIO.2004.01.004) 15142756

[47] Nickerson DP, Buist ML 2009 A physiome standards-based model publication paradigm. Phil. Trans. R. Soc. A 367, 1823–1844. (10.1098/rsta.2008.0296) 19380314

[48] Garny A, Nickerson DP, Cooper J, Santos RWD, Miller AK, McKeever S, Nielsen P MF, Hunter PJ 2008 Cell ML and associated tools and techniques. Phil. Trans. R. Soc. A 366, 3017–3043. (10.1098/rsta.2008.0094) 18579471

[49] Nikishova A, Veen L, Zun P, Hoekstra AG 2018 Uncertainty quantification of a multiscale model for in-stent restenosis. Cardiovasc. Eng. Technol. 9, 1–14. (10.1007/s13239-018-00372-4) 30136082 PMC6290695

[50] Neumann P, Bian X 2017 MaMiCo: Transient multi-instance molecular-continuum flow simulation on supercomputers. Comput. Phys. Commun. 220, 390–402. (10.1016/J.CPC.2017.06.026)

[51] Jacob CR, Beyhan SM, Bulo RE, Gomes ASP, Götz AW, Kiewisch K, Sikkema J, Visscher L 2011 PyADF - A scripting framework for multiscale quantum chemistry. J. Comput. Chem. 32, 2328–2338. (10.1002/jcc.21810) 21541961

[52] Alowayyed S, Závodszky G, Azizi V, Hoekstra AG 2018 Load balancing of parallel cell-based blood flow simulations. J. Comput. Sci. 24, 1–7. (10.1016/J.JOCS.2017.11.008)

[53] Bhati AP, Wan S, Wright DW, Coveney PV 2017 Rapid, accurate, precise, and reliable relative free energy prediction using ensemble based thermodynamic integration. J. Chem. Theory Comput. 13, 210–222. (10.1021/acs.jctc.6b00979) 27997169

[54] Coveney PV, Wan S 2016 On the calculation of equilibrium thermodynamic properties from molecular dynamics. Phys. Chem. Chem. Phys. 18, 30 236–30 240. (10.1039/C6CP02349E) 27165501

